# Neurodegeneration in the cortical sulcus is a feature of chronic traumatic encephalopathy and associated with repetitive head impacts

**DOI:** 10.1007/s00401-024-02833-8

**Published:** 2024-12-06

**Authors:** Raymond Nicks, Arsal Shah, Spiro Anthony Stathas, Daniel Kirsch, Sarah M. Horowitz, Nicole Saltiel, Samantha M. Calderazzo, Morgane L. M. D. Butler, Kerry A. Cormier, Nurgul Aytan, Fatima Tu-Zahra, Rebecca Mathias, Farwa Faheem, Suzie Marcus, Elizabeth Spurlock, Lucas Fishbein, Camille D. Esnault, Alexandra Boden, Grace Rosen, Weiming Xia, Sarah Daley, Gaoyuan Meng, Brett R. Martin, Daniel H. Daneshvar, Christopher J. Nowinski, Michael L. Alosco, Jesse Mez, Yorghos Tripodis, Bertrand R. Huber, Victor E. Alvarez, Jonathan D. Cherry, Ann C. McKee, Thor D. Stein

**Affiliations:** 1 Boston University Alzheimer’s Disease and CTE Center, Boston Chobanian & Avedisian University School of Medicine, Boston, MA USA; 2 VA Boston Healthcare System, Boston, MA USA; 3 Department of Neurology, Boston University Chobanian & Avedisian School of Medicine, Boston, MA USA; 4 VA Bedford Healthcare System, Bedford, MA USA; 5 Department of Biostatistics, Boston University School of Public Health, Boston, MA USA; 6 Department of Pathology and Laboratory Medicine, Boston University School of Medicine, Boston, MA USA; 7 Department of Physical Medicine and Rehabilitation, Harvard Medical School, Boston, MA USA; 8 Department of Physical Medicine and Rehabilitation, Massachusetts General Hospital, Boston, MA USA; 9 Department of Physical Medicine and Rehabilitation, Mass General Brigham-Spaulding Rehabilitation, Charlestown, MA USA; 10 Concussion Legacy Foundation, Boston, MA USA; 11 Department of Anatomy & Neurobiology, Boston University Chobanian & Avedisian School of Medicine, Boston, MA USA; 12 Department of Biological Sciences, Kennedy College of Science, University of Massachusetts, Lowell, MA USA

**Keywords:** Chronic traumatic encephalopathy, Repetitive head impacts, Contact sports, Neurodegeneration, Cortical thinning, Neuronal loss, Synaptic loss, Cortical sulcus, Tau pathology

## Abstract

Neurodegeneration is a seminal feature of many neurological disorders. Chronic traumatic encephalopathy (CTE) is caused by repetitive head impacts (RHI) and is characterized by sulcal tau pathology. However, quantitative assessments of regional neurodegeneration in CTE have not been described. In this study, we quantified three key neurodegenerative measures, including cortical thickness, neuronal density, and synaptic proteins, in contact sport athletes (*n* = 185) and non-athlete controls (*n* = 52) within the sulcal depth, middle, and gyral crest of the dorsolateral frontal cortex. Cortical thickness and neuronal density were decreased within the sulcus in CTE compared to controls (*p*’s < 0.05). Measurements of synaptic proteins within the gyral crest showed a reduction of α-synuclein with CTE stage (*p* = 0.002) and variable changes in PSD-95 density. After adjusting for age, multiple linear regression models demonstrated a strong association between the duration of contact sports play and cortical thinning (*p* = 0.001) and neuronal loss (*p* = 0.032) within the sulcus. Additional regression models, adjusted for tau pathology, suggest that within the sulcus, the duration of play was associated with neuronal loss predominantly through tau pathology. In contrast, the association of duration of play with cortical thinning was minimally impacted by tau pathology. Overall, CTE is associated with cortical atrophy and a predominant sulcal neurodegeneration. Furthermore, the duration of contact sports play is associated with measures of neurodegeneration that are more severe in the cortical sulcus and may occur through tau-dependent and independent mechanisms.

**Supplementary Information:**

The online version contains supplementary material available at 10.1007/s00401-024-02833-8.

## Introduction

Chronic traumatic encephalopathy (CTE) is a progressive neurodegenerative disease caused by repetitive head impacts (RHI), typically sustained through playing contact or collision sports, including American football, ice hockey, boxing, soccer, or rugby [10, 11, 50, 52, 53]. Clinical symptoms of CTE include impairments in cognition, behavior, mood, and motor functioning and typically manifest years or decades after exposure to RHI [56, 58, 70]. Gross neuropathological changes in CTE include prominent frontal lobe atrophy as well as temporal lobe, thalamic, and mammillary body atrophy in higher stage disease [47, 50, 53]. CTE is defined neuropathologically by the perivascular accumulation of abnormally hyperphosphorylated tau in neurons and occasionally in astrocytes with a predilection for the depths of cortical sulci [4, 10, 48]. Computational modeling of head impacts demonstrates that strain forces concentrate at convexities of the brain, such as sulcal depths, and blood vessels, potentially explaining the pattern of pathology observed in CTE [27, 30, 83].

Neurodegeneration, including cortical thinning and synaptic and neuronal loss, is a prominent feature of tauopathies. For instance, in Alzheimer’s disease (AD), numerous studies have demonstrated cortical thinning [25, 32, 62], neuronal loss [21], and synaptic loss [23, 24, 41, 44, 64, 65] within the medial temporal lobe and neocortical regions [9, 22, 78]. Measures of synaptic proteins are also decreased in frontotemporal lobar degenerations [12, 20]. However, quantitative assessments of neurodegeneration in CTE are lacking and have not considered the regional involvement of the cortical sulcus.

Here, we quantify neurodegeneration in CTE by measuring changes in gray matter cortical thickness, neuronal density, and synaptic protein levels. We hypothesized that greater neurodegeneration will be present at the depths of the sulcus and with increasing CTE stage. Using the duration of contact sports play as a proxy for cumulative RHI exposure, we also test the hypothesis that years of play would predict increased neurodegeneration in CTE.

## Materials and methods

### Participants

Participants were selected from two different brain donor groups, including the Understanding Neurologic Injury and Traumatic Encephalopathy (UNITE) study that includes those with a history of RHI through contact/collision sports exposure such as football, ice hockey, boxing, soccer, rugby, and martial arts [57] and the Framingham Heart Study (FHS), a longitudinal and community-based study. Neuropathological processing included comprehensive screening for neurodegenerative conditions following procedures established for the UNITE brain bank [57, 76]. Participants with the following comorbidities were excluded: intermediate or high Alzheimer’s disease by NIA-Reagan criteria, motor neuron disease (MND), frontotemporal lobar degeneration (both FTLD-TDP and FTLD-tau, including corticobasal degeneration [CBD] and progressive supranuclear palsy [PSP]), neocortical Lewy body disease (LBD), and primary brain neoplasms. Age-related pathologies, including primary age-related tauopathy (PART) and limbic-predominant TDP-43 inclusions, were not excluded in order to increase the generalizability of the study. Of the 1109 participants from UNITE and FHS who had tissue available prior to February 24, 2020, 316 participants met inclusion criteria and had available fresh frozen dorsolateral frontal cortex tissue. Since 97% of the participants who had repetitive head impacts were male, we did not include 79 female controls, leaving our final dataset with *n* = 237 participants. These participants were broken down into four groups: control (*n* = 52), RHI (*n* = 48), Low CTE (*n* = 49), and High CTE (*n* = 88).

All brains were fully assessed for gross and microscopic pathology by neuropathologists (AM, TS, VA, BH) blinded to the clinical evaluation. The brains were hemisected, and pathological assessment was performed on the fixed hemisphere, while the opposing hemisphere was frozen for biochemical protein quantification. Atrophy patterns were graded with a four-point scale including none (0), mild (1), moderate (2), and severe (3) atrophy using criteria from the National Alzheimer’s Coordinating Center (NACC) neuropathology assessment. Subcortical atrophy was determined as present or absent, and the presence of a thalamic notch was defined as medial thalamic atrophy that results in a sharp curvature of the thalamus [51]. Such gross pathology measures have been shown to correlate with clinical symptoms [80]. CTE was diagnosed based on consensus criteria [10, 48]. Staging was based on regional p-tau involvement according to the McKee staging system since this has been shown to correlate with duration of play and clinical symptoms [4, 53]. Stages were then dichotomized into low (I and II) and high (III and IV) stages, which shows good agreement with low and high stages using consensus criteria [4, 53]. Participants with no evidence of CTE and without RHI exposure, were labeled the “control” group. Participants who had no evidence of CTE but had RHI exposure were labeled the “RHI” group. Participants diagnosed with CTE Stage I or II were grouped as “Low CTE.” Lastly, participants diagnosed with CTE Stage III or IV were grouped as “High CTE.” There were no cases of CTE without a history of RHI.

Next of kin and other informants of the brain donors in both UNITE and FHS completed the Boston University Repetitive Head Impact Exposure Assessment (BU-RHIEA) questionnaire to determine RHI status and duration of contact sports play [14]. The duration of contact sports play was used as a proxy for cumulative RHI exposure as previously described [55]. Total years of all contact sports participation, including American football, was determined from next-of-kin after death and by checking with an online database for professional players [55] in the UNITE and FHS study groups. For participants who played multiple sports, the primary sport was determined by the highest level of play (e.g., professional, semi-professional, college, high school, and youth). If a participant played both sports at the same level, the sport with the longer duration was determined as their primary sport. Next-of-kin provided written consent for research participation and brain donation. Institutional review boards of the Boston University Medical Center and the Bedford Veteran’s Affairs (VA) Healthcare System approved all study protocols.

### Histochemistry and immunohistochemistry

All brain tissue was processed identically by fixation in periodate-lysine-paraformaldehyde and stored at 4 °C. Multiple tissue blocks were taken, including from the dorsolateral frontal cortex (DLFC) perpendicular to the superior frontal sulcus, embedded in paraffin, and cut at 10 μm. The sections of paraffin-embedded tissue from the DLFC were stained for hyperphosphorylated tau (AT8; Invitrogen MN1020; 1:1000), NeuN (BioLegend; 1:750), and luxol fast blue, and hematoxylin and eosin (LHE), using previously described methods [49]. Stained slides were scanned at 20 × magnification with a Leica Aperio Scanscope (Leica Biosystems, Richmond, IL). Slides were examined using the Aperio eSlide Manager (Leica Biosystems) and the Aperio ImageScope (Leica Biosystems) software.

Gray matter cortical thickness was measured by using the ImageScope ruler tool on LHE stained sections. Cortical thickness was calculated by measuring three lines orthogonal to the pial surface and then computing the average. The average gray matter cortical thickness was calculated at the depth of the cortical sulcus (defined as the bottom third of two connecting gyri), in the middle (defined as the middle third of two connecting gyri), and at the gyral crest (defined as the top third of two connecting gyri). In the sulcus and middle, sample sizes were 40 for control, 31 for RHI, 34 for Low CTE, and 54 for High CTE due to missing data. In the crest, sample sizes were 39 for control, 31 for RHI, 34 for Low CTE, and 54 for High CTE. The reasons for missingness included poor tissue quality or incomplete brain specimens that excluded the area of analysis. Group sizes were based on available tissue and not reduced to match between analyses to prevent loss of statistical power.

Neuronal density was quantified by using ImageScope (Leica Biosystems). The gray matter was highlighted from the pia to the boundary between the white and gray matter. NeuN has previously been used as a reliable tool for the quantitative study of neuronal morphometry in postmortem human brain tissue [31]. Therefore, neuronal density was quantified by Leica’s image analysis and automated counting software (Aperio nuclear algorithm, Version 9, Leica Biosystems) that identified cells positively labeled for NeuN within the gray matter. The total number of NeuN cells was divided by the area of analysis to determine the NeuN cell density. NeuN density was measured in the sulcus, middle, and crest. In the sulcus, sample sizes were reduced to 26 for control, 20 for RHI, 19 for Low CTE, and 36 for High CTE. In the middle and crest, sample sizes were reduced to 26 for control, 19 for RHI, 15 for Low CTE, and 30 for High CTE. Neuronal density quantification was not performed if the stain failed due to excessive fixation, there was poor tissue quality, the tissue source was exhausted, or the region for analysis was absent. NeuN staining is sensitive to fixation and, therefore, the sample size was reduced compared to other measures.

For tau density, Leica’s image analysis and automated counting software (Aperio nuclear algorithm, Version 9, Leica Biosystems) was calibrated for shape, size, and staining intensity to detect AT8-immunoreactive neurofibrillary tangles (NFTs) within the gray matter. Counts were normalized to the area measured and are presented as density within the analyzed region. For both NeuN and NFT counts, all identified objects were manually checked and any flagged cases as well as a random subset were further inspected by a neuropathologist (TDS) or trained morphologist (JDC) to validate that the algorithm was correctly identifying either NeuN+ cells or AT8+ neuronal tangles. Mis-identified object were re-classified and analyzed again. Astrocytic tau tangles were generally smaller, lacked a pyramidal shape, and were largely excluded from the analysis.

### Quantitative immunoassay measurements of α-synuclein and PSD-95

Frozen tissue from the gyral crest of the dorsolateral prefrontal cortex was weighed and placed on dry ice. Brain tissue was homogenized in a 5:1 volume of freshly prepared, ice-cold 5 M Guanidine Hydrochloride in Tris-buffered saline (20 mM Tris–HCl, 150 mM NaCl, pH 7.4), which contained 1:100 Halt protease inhibitor cocktail (Thermo Fischer Scientific, Waltham, MA) and 1:100 Phosphatase inhibitor cocktail 2 & 3 (Sigma-Aldrich, St. Louis, MO) as previously reported [68, 69]. The homogenate was then shaken (regular rocker) overnight at room temperature. The lysate was diluted with 1% Blocker A (Meso Scale Discovery (MSD), Rockville, Maryland, #R93BA-4) in wash buffer according to specific immunoassays: 1:4000 for α-synuclein (MSD #K151WKK-2) and 1:3000 for postsynaptic density protein-95 (PSD-95, MSD #K250QND). Samples were centrifuged at 17,000 g and 4 °C for 15 min. The supernatant was subsequently applied to the immunoassays, and the original homogenate was aliquoted and stored at − 80 °C. Relative PSD-95 units were calculated based on a standard curve for a reference brain lysate. Standards with known concentrations were used for α-synuclein, and all standards and samples were run in duplicate. Measurements were made using the multi-detection SPECTOR 2400 Imager (MSD). The sample size for α-synuclein was 48 for control, 45 for RHI, 42 for Low CTE, and 80 for High CTE. The sample size for PSD-95 was 49 for control, 46 for RHI, 45 for Low CTE, and 80 for High CTE. Sample sizes were reduced due to missing data or exhausted tissue sources.

### Statistical methodology

Statistical analysis was performed using SPSS 27.0 (IBM Corp) and Prism v9 (GraphPad Software). The means were compared between all four groups (control, RHI, Low CTE, High CTE) using the Kruskal–Wallis test, and values were adjusted by Bonferroni correction for multiple tests for continuous and ordinal variables. The chi-square test was used for proportions to evaluate dichotomous variables. An analysis of covariance (ANCOVA) was used to compare differences between the pathology groups for cortical thickness, neuronal density, and synaptic protein density measurements, adjusting for age at death and postmortem interval (PMI). Gray matter cortical thickness measurements, as well as α-synuclein and PSD-95 concentrations, underwent rank-based normalization for regression analyses [71]. Multiple linear regression analyses were used to evaluate associations between the dependent variables (cortical thickness, neuronal density, and synaptic protein density) and predictors, total years of contact sports play and tau pathology (AT8), adjusting for age at death and PMI. For a final model, we employed a mediation analysis technique [34] to examine the direct and indirect effects of the independent variables (RHI and age) on the dependent variables NeuN density and tau pathology (AT8) within the frontal sulcus. Parameter estimates were used to quantify the strength and significance of the mediated pathway on the total sample of participants with a history of repetitive head impacts. Statistical significance throughout was set as 0.05.

## Results

### Demographic differences

Based on RHI exposure history and the presence of CTE, participants were grouped as control, RHI, Low CTE, or High CTE (Table 1). There was no significant difference in race. Age at death and postmortem interval (PMI) differed significantly between groups, and these variables were controlled for in subsequent analyses. Those with a history of RHI were also significantly more likely to have had a traumatic brain injury (TBI) with a loss of consciousness (LOC) compared to controls (Table 1). In the control group, six participants reported a history of TBI with LOC resulting from diverse causes, including significant falls, motor vehicle accidents, or military blast exposure. In contrast, nearly all of those with a history of RHI sustained a TBI with LOC from contact sports (> 90%).

**Table 1:** Demographic and contact sports exposure of pathological groups

Characteristic	Control (%) (*n* = 52)	RHI (%) (*n* = 48)	Low CTE (%) (*n* = 49)	High CTE (%) (*n* = 88)	*p*-value
**Cohort**					
UNITE	0	100% (48)	100% (49)	100% (88)	
FHS	100% (52)	0	0	0	
**Race**					**0.508**
White	42.3% (22)	87.5% (42)	83.7% (41)	88.6% (78)	
Black/African American	0	10.4% (5)	12.3% (6)	11.4% (10)	
Other	0	2.1% (1)	2% (1)	0	
Unknown	57.7% (30)	0	2% (1)	0	
**Age at death, years**	82.4 (1.53)	52.8 (3.26)	55.0 (2.49)	73.5 (1.34)	< 0.001*^a^^b^^c^^e^^f^
Range	53–96	14–88	25–84	32–97	
**PMI, hours**	24.6 (3.6)	43.8 (3.5)	42.9 (3.2)	34.3 (2.2)	< 0.001*^a^^b^^c^^e^^f^
**Duration of contact sports play, years**	0	10.7 (1.0)	15.1 (1.3)	17.0 (0.7)	< 0.001*^a^^b^^c^^d^^e^
**Main sport played, % (*n*)**					
American Football	0	85.4% (41)	86% (42)	94.3% (83)	
Ice Hockey	0	2.1% (1)	2% (1)	3.4% (3)	
Boxing	0	2.1% (1)	2% (1)	2.3% (2)	
Other^g^	0	10.4% (5)	10% (5)	0	
**History of TBI**					
TBI with LOC % (*n*)	17.1% (6)	56.8% (25)	70.4% (31)	65.8% (52)	< 0.001*^a^^b^^c^

Data are presented as mean (SEM) for continuous variables and % (n) for categorical variables. *p < 0.05, Analysis of Variance with post hoc least significant difference statistical testing *p* < 0.05 as follows:

^a^RHI vs. control

^b^Low CTE vs. control

^c^High CTE vs. control

^d^Low CTE vs. RHI

^e^High CTE vs. RHI

^f^High CTE vs. Low CTE

^g^Includes soccer, rugby, amateur wrestling, martial arts, and karate

For PMI, *N* = 220; main sport played, *N* = 185; duration of contact sports play, *N* = 235; TBI with LOC, *N* = 202

*CTE* chronic traumatic encephalopathy, *FHS* framingham heart study, *LOC* loss of consciousness, *PMI* post-mortem interval, *RHI* repetitive head impacts, *SEM* standard error of mean, *TBI* traumatic brain injury, *UNITE* understanding neurologic injury and traumatic encephalopathy

### Gross anatomical changes

Atrophy patterns were significantly different between groups (Table 2). High CTE demonstrated greater atrophy across all cortical and hippocampal regions compared to control, RHI, and Low CTE groups (*p*’s < 0.05). Atrophy scores were greatest in the frontal lobe and hippocampus in High CTE. There were no significant differences in lobar atrophy scores between control, RHI, and Low CTE groups. In addition, High CTE had a greater frequency of atrophy noted in the hypothalamus and mammillary body as well as a greater proportion of cases with a thalamic notch (indicative of thalamic atrophy) compared to the other groups (Table 2; *p*’s < 0.05).

**Table 2:** Gross pathological changes

Characteristic	Control (*n* = 52)	RHI (*n* = 48)	Low CTE (*n* = 49)	High CTE (*n* = 88)	*p*-value
**Atrophy, 0–3, mean (SEM)**					
Frontal lobe	0.73 (0.117)	0.72 (0.134)	0.69 (0.110)	1.83 (0.097)	< 0.001*^a^^b^^c^
Temporal lobe	0.42 (0.088)	0.50 (0.119)	0.50 (0.103)	1.65 (0.098)	< 0.001*^a^^b^^c^
Parietal lobe	0.25 (0.061)	0.35 (0.099)	0.31 (0.090)	1.13 (0.102)	< 0.001*^a^^b^^c^
Occipital lobe	0.13 (0.048)	0.13 (0.067)	0.13 (0.057)	0.58 (0.094)	< 0.001*^a^^b^^c^
Total cortical	1.29 (0.221)	0.65 (0.125)	0.60 (0.106)	1.71 (0.096)	< 0.001*^a^^c^
Hippocampus	0.79 (0.187)	0.48 (0.103)	0.62 (0.106)	1.87 (0.104)	< 0.001*^a^^b^^c^
**Subcortical atrophy, % present (present/absent)**					
Hypothalamus	0.0% (0/13)	17.1% (7/34)	21.7% (10/36)	62.4% (53/32)	< 0.001*^a^^b^^c^
Mamillary body	0.0% (0/13)	16.7% (7/35)	23.9% (11/35)	61.0% (50/32)	< 0.001*^a^^b^^c^
Thalamic notch	7.7% (1/12)	11.9% (5/37)	25.0% (11/33)	47.7% (41/45)	< 0.001*^a^^b^
**Septum pellucidum abnormalities, % present (present/absent)**					
Septum cavum	10.0% (1/9)	16.7% (6/30)	33.3% (11/22)	45.6% (26/31)	0.012*^c^
Septal fenestrations	0.0% (0/10)	0.0% (0/36)	21.2% (7/26)	47.2% (25/28)	< 0.001*^c^

Data are presented as mean (SEM) for continuous variables and % (*n*) for categorical variables. *p < 0.05, Analysis of Variance with post hoc least significant difference statistical testing *p* < 0.05 as follows:

^a^High CTE vs. control

^b^High CTE vs. RHI

^c^High CTE vs. low CTE

For Septum cavum, *N* = 136; Septal fenestrations, *N* = 136; Thalamic notch, *N* = 185; Hypothalamus, *N* = 185; Mamillary bodies, *N* = 181; Total cortical, *N* = 194

*CTE* chronic traumatic encephalopathy, *RHI* repetitive head impacts, *SEM* standard error of mean

Abnormalities of the septum pellucidum were also frequent in CTE. Notably, High CTE had a higher frequency of a cavum septum pellicidum than the RHI group (*p* < 0.05). Both Low CTE and High CTE were more likely to have septum fenestrations compared to control and RHI groups (*p*’s < 0.05).

### Associations between RHI exposure and gray matter cortical thickness

We examined the burden of tau pathology across multiple cortical regions. The tau pathology burden increased with RHI and CTE across regions in the frontal, temporal, and parietal lobes with the greatest burden in the middle frontal region (Supplementary Table 1, online resource). We, therefore, focused on the dorsolateral middle frontal cortex for the remainder of the study.

**Fig. 1:**
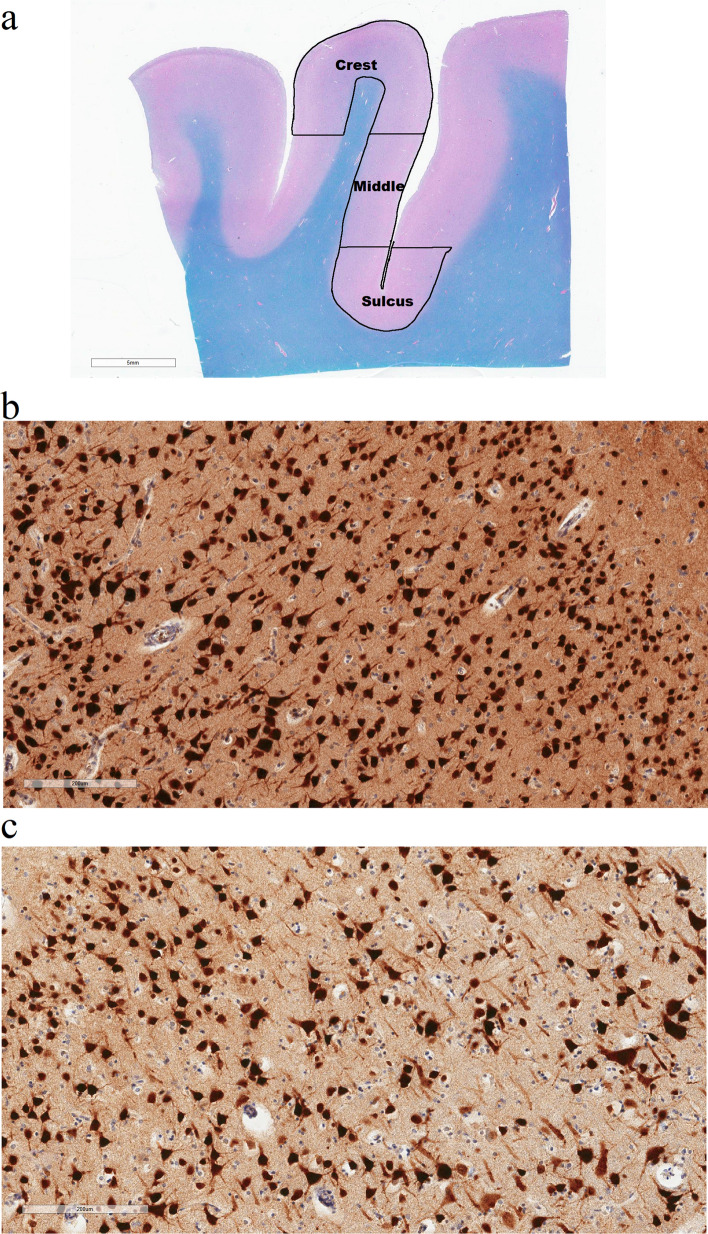
Representative images of NeuN staining in CTE. **a** Cortical thickness was calculated by measuring three lines orthogonal to the pial surface and then computing the average. Analysis was based on three areas: crest, middle, and sulcus. **b**, **c** The number of NeuN+ cells in the sulcus of the dorsolateral frontal cortex was greater in control (**b**) compared to High CTE (**c**). Scale bars: **a** 5 mm; **b**, **c** 200 μm

**Fig. 2:**
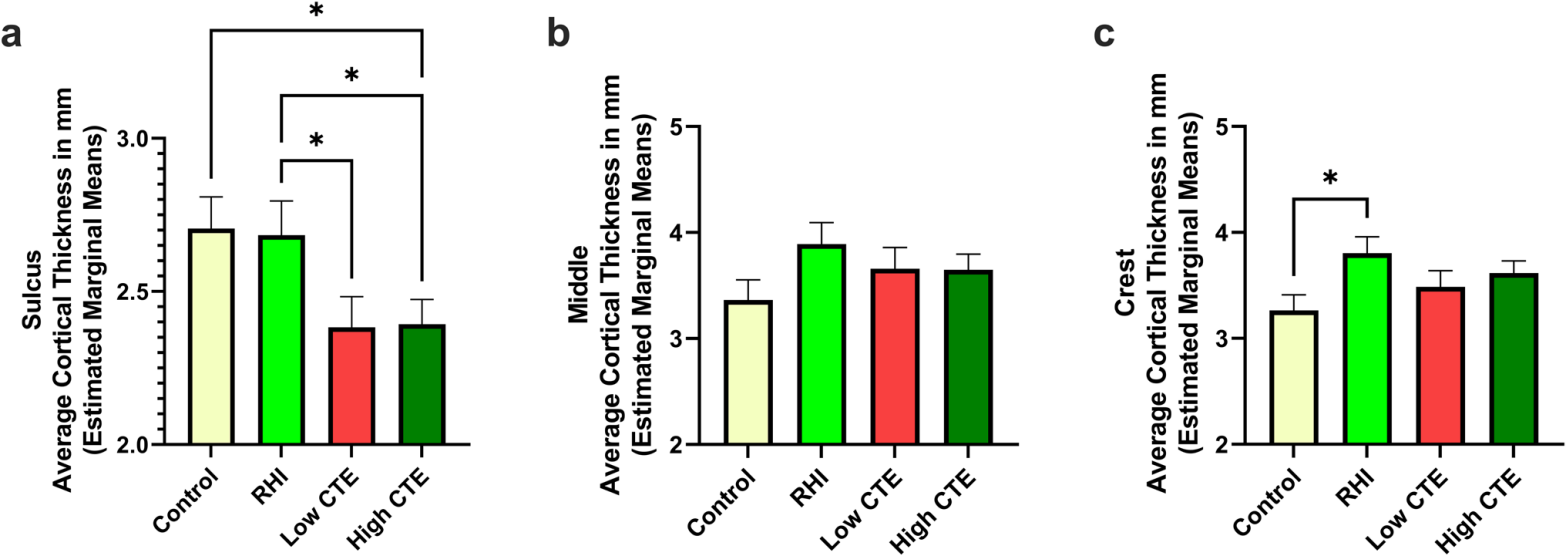
Cortical thickness measurements by pathology group in dorsolateral frontal gray matter in **a** sulcus, **b** middle, and **c** crest. **a** Cortical thickness within the sulcus was significantly different between groups, as shown by ANCOVA (*p* = 0.008). Post hoc pairwise comparisons showed that High CTE was significantly less than control (*p* = 0.015) and RHI (*p* = 0.04), and Low CTE was reduced compared to the RHI group (*p* = 0.039). **b** Within the middle of the gyrus, there were no significant differences between groups. **c** At the gyral crest, the cortical thickness was significantly different between groups, as shown by ANCOVA (*p* = 0.01). Post hoc pairwise comparison showed that the RHI group was significantly greater than control (*p* = 0.021). *p < 0.05, analysis of covariance adjusting for age at death and PMI

Cortical thickness measurements were conducted along the superior frontal sulcus with the dorsolateral frontal cortex (DLFC) divided into three regions of interest: sulcus, middle, and crest (Fig. 1a). After adjusting for age and PMI, the greatest differences between groups were seen within the DLFC sulcus (Fig. 2a, p = 0.008), with reduced cortical thickness in both Low CTE and High CTE compared to the RHI group (*p*’s < 0.05) as well as reduced cortical thickness in High CTE compared to the control group (*p* = 0.015). No significant differences were found among the groups in the middle third of the gyrus (Fig. 2b). In the gyral crest, the RHI group showed increased cortical thickness compared to the control group (*p* = 0.021, Fig. 2c). Comparison of non-age adjusted cortical thickness also showed significant decreases in the sulcus within High CTE compared to control (*p* = 0.022) and RHI (*p* = 0.004) groups (Supplementary Figure a, online resource). To examine differences within individuals, we calculated the ratio of the cortical thickness at the sulcus to the thickness in the gyral crest and found that RHI (*p* = 0.004), Low CTE (*p* = 0.001), and High CTE (*p* < 0.001) groups all had lower sulcus/crest ratios than the control group (Supplementary Figure b, online resource).

Multiple linear regression analyses were run in the sulcus, middle, and crest to determine potential associations between duration of contact sports play and cortical thickness, adjusting for age at death and PMI (Table 3a). There was a significant negative association between the duration of contact sports play and cortical thickness in the sulcus (*β* = −0.254, *p* = 0.001), but not in the middle third of the gyrus or in the gyral crest. Because duration of play is associated with tau pathology, which may partially mediate cortical thinning, a second multiple linear regression tested associations between cortical thickness and duration of contact sports play while adjusting for tau pathology (Table 3b). This analysis demonstrated that both years of contact sports play and tau pathology (AT8 density at the sulcus) were significant predictors for reduced cortical thickness in the sulcus. To determine if the primary sport of participation had an effect, we employed a sensitivity analysis consisting of participants who had a history of American football play. The results were similar and showed a significant association between the duration of football participation and reduced cortical thickness in the sulcus (*β* = −0.200, *p* = 0.012). An additional regression was run adjusting for a history of TBI with a loss of consciousness, and the results were similar without a significant contribution from TBI (duration of play: *β* = −0.248, *p* = 0.005; TBI: *β* = 0.026, *p* = 0.756). Within a subset of CTE cases (*N* = 22), 13 (59%) had the CTE pathognomonic perivascular tau pathology lesion within the dorsolateral frontal cortex section examined.

**Table 3:** Multiple linear regression analyses modelling cortical thickness with duration of contact sports play in the sulcus, middle, and crest of dorsolateral frontal cortex

a) Associations with cortical thickness in the sulcus, middle, and crest						
Predictor	Sulcus		Middle		Crest	
	*β*	*p*-value	β	*p*-value	β	*p*-value
Duration of contact sports play (years)	**−0.254**	**0.001**	0.074	0.347	0.081	0.296

b) Associations with cortical thickness in the sulcus, adjusting for tau pathology		
Predictor	Sulcus	
	*β*	*p*-value
Duration of contact sports play (years)	**−0.227**	**0.013**
Tau pathology (AT8) sulcus	**−0.304**	** < 0.001**

a) Multiple linear regressions were run in the sulcus, middle, and crest to determine associations between cortical thickness (dependent variable) and total years of playing contact sports. b) A second multiple linear regression model was run in the sulcus to determine associations between cortical thickness and total years of playing contacts sports, adjusting for tau pathology. The cortical thickness variables displayed for the sulcus, middle, and crest underwent rank-based normalization, and both models were adjusted for age at death and post-mortem interval. *β* indicates standardized beta value. Significant associations are shown in bold

### Associations between RHI exposure and neuronal density

**Fig. 3:**
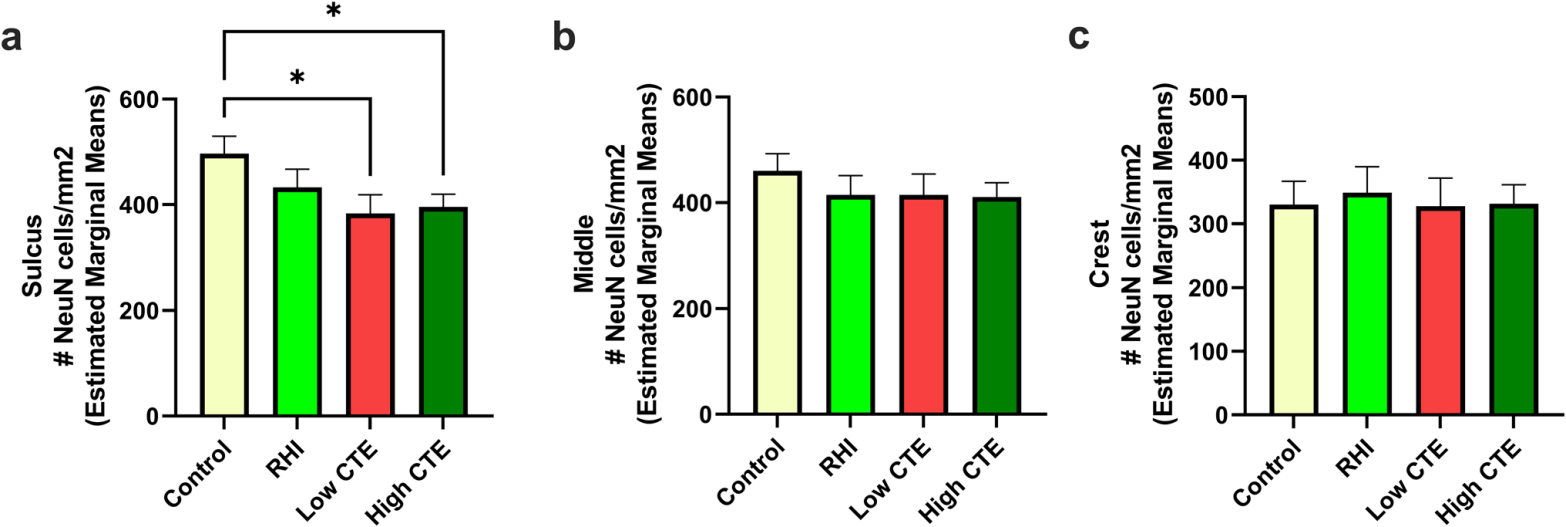
Neuronal density by pathology group in dorsolateral frontal gray matter in **a** sulcus, **b** middle, and **c** crest. **a** Neuronal density within the sulcus was significantly different between groups, as shown by ANCOVA (*p* = 0.05). Post hoc pairwise comparisons showed that neuronal density was reduced compared to the control group in both Low CTE (*p* = 0.03) and High CTE (*p* = 0.014). **b** Within the middle of the gyrus there were no significant differences between groups. **c** At the gyral crest, there were no significant differences between groups. *p < 0.05, analysis of covariance adjusting for age at death and PMI

In order to examine whether the reduction in cortical thickness might be partially due to decreased neuronal density, we examined associations with NeuN+ cellular density in the sulcus, middle, and gyral crest. Within the sulcus, there was significantly lower neuronal density in both Low CTE and High CTE groups compared to the control group (*p*’s < 0.05, Fig. 3a). In both the middle and gyral crest regions, no significant differences in neuronal density were observed among the groups (Fig. 3b and Fig. 3c). Immunohistochemical staining for NeuN illustrates the decreased neuronal density within the sulcus in High CTE (Fig. 1c) compared to control (Fig. 1b).

Multiple linear regression analyses tested for associations between duration of contact sports play and neuronal density, adjusting for age at death and PMI (Table 4). There was a significant negative association between the duration of contact sports play and neuronal density in the sulcus (*β* = −0.231, *p* = 0.032), but the association with neuronal density in the middle third and gyral crest were not significant (Table 4a). In order to test whether tau pathology might partially underlie the decreased neuronal density within the sulcus, a second multiple linear regression model included levels of AT8+ tau pathology within the sulcus. Sulcal tau pathology, but not duration of play, significantly predicted decreased neuronal density (*β* = −0.344, *p* = 0.007, Table 4b), suggesting that duration of play leads to decreased neuronal density through tau pathology within the sulcal depths. A sensitivity analysis restricted to football players also showed an association between the duration of football participation and decreased neuronal density in the sulcus (*β* = −0.229, *p* = 0.035).

**Table 4:** Multiple linear regression analyses modelling neuronal density (NeuN/mm^2^) with duration of contact sports play in the sulcus, middle, and crest of dorsolateral prefrontal cortex

a) Associations with neuronal density in the sulcus, middle, and crest						
Predictor	Sulcus		Middle		Crest	
	*β*	*p*-value	*β*	*p*-value	*β*	*p*-value
Duration of contact sports play (years)	**−0.231**	**0.032**	−0.195	0.064	−0.130	0.287

b) Associations with neuronal density in the sulcus, adjusting for tau pathology		
Predictor	Sulcus	
	*β*	*p*-value
Duration of contact sports play (years)	−0.174	0.219
Tau pathology (AT8) sulcus	**−0.344**	**0.007**

a) Multiple linear regressions were run in the sulcus, middle, and crest to determine associations between neuronal density and total years of playing contact sports. b) A second multiple linear regression model was run in the sulcus to determine associations between neuronal density and total years of playing contact sports, adjusting for tau pathology. Both models were adjusted for age at death and post-mortem interval. *β* indicates standardized beta value. Significant associations are shown in bold

Although participants with significant other pathologies including AD or FTLD were excluded from this study, low level pathologies might contribute to neurodegeneration. Therefore, sensitivity analyses were performed for additional pathologies that might contribute to cortical thinning and neuronal loss, including neuritic beta-amyloid plaques, AD-type tau pathology, and TDP-43 inclusions. Separate multiple linear regressions showed that together with tau pathology in the model neither neuritic beta-amyloid plaque score nor Braak stage significantly contributed to cortical thickness or neuronal density at the cortical sulcus. The presence of TDP-43 was significantly associated with cortical thickness (*β* = −0.353, *p* = 0.002), but not neuronal density.

### CTE-associated loss of synaptic proteins

**Fig. 4:**
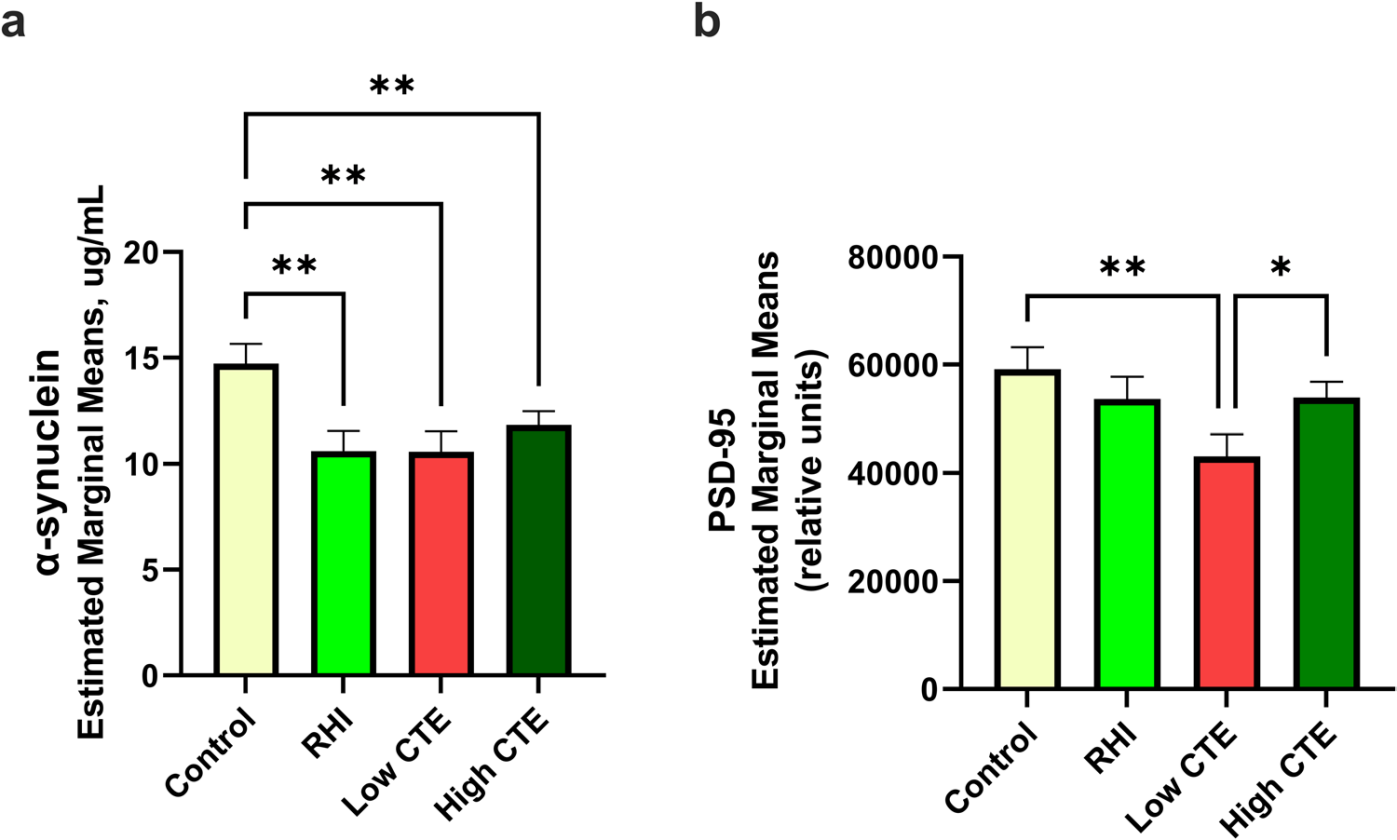
α-synuclein and PSD-95 concentrations as measures of synaptic density in dorsolateral frontal gray matter tissue. **a** α-synuclein levels were significantly different between groups, as shown by ANCOVA (*p* = 0.028). Post hoc pairwise comparisons showed that RHI, Low CTE, and High CTE group all had significantly less α-synuclein than the control group (*p* < 0.01). **b** PSD-95 levels were significantly different between groups, as shown by ANCOVA (*p* < 0.001). Post-hoc pairwise comparisons showed that the Low CTE group had less PSD-95 than the control group (*p* = 0.009) and the High CTE group was increased as compared to Low CTE (*p* = 0.039). *p < 0.05 and **p < 0.01, analysis of covariance adjusting for age at death and PMI

Synaptic protein levels were quantified by using immunoassays to assess the concentration of α-synuclein (presynaptic) and PSD-95 (postsynaptic) in the gyral crest of the DLFC gray matter tissue. Levels of α-synuclein have been shown to decrease in AD and to decrease with the duration of dementia [75]. PSD-95 served as a postsynaptic marker [33, 36]. RHI, Low CTE, and High CTE groups all showed significantly lower α-synuclein density compared to the control group (*p*’s < 0.01, Fig. 4a). There was significantly decreased PSD-95 density in Low CTE compared to the control group (*p* = 0.009), but paradoxically increased PSD-95 density in High CTE compared to Low CTE (*p* = 0.039, Fig. 4b). Previous studies have shown increased variability and altered distributions in dendritic/spine measures [77], and a dynamic damage and repair process might explain the increased PSD-95 density in High CTE. In order to further test whether variation in synaptic proteins are higher with RHI and CTE, we calculated the coefficient of variation (CV = [standard deviation/mean] × 100) for both α-synuclein and PSD-95 protein densities. For α-synuclein, CVs were slightly higher in RHI (49.9%), Low CTE (52.3%), and High CTE (48.3%) than in the control (45.7%) group. Similarly, for PSD-95, CVs were higher in the RHI (54.2%), Low CTE (50.1%), and High CTE (57.5%) groups than in the control (47.7%) group.

### Pathway analysis of the effects of RHI on neuronal density within the sulcus

**Fig. 5:**
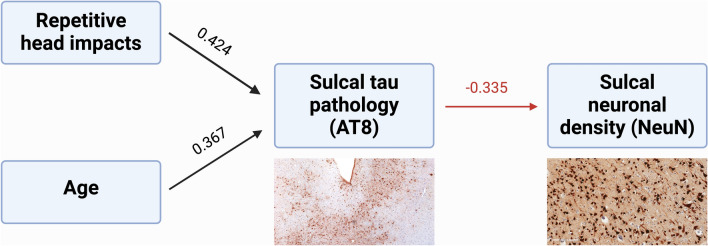
Schematic representation of mediation analysis modeling associations between repetitive head impacts, age of death, tau pathology, and neuronal density in the sulcus. Black arrows represent significant positive associations, and the red arrow represents significant negative association (*p*’s < 0.001). Standardized *β*’s are shown above arrows. Created in BioRender. Stein, T. (2024) BioRender.com/h62i404

For a final model, we employed a mediation analysis, with neuronal density and tau pathology (AT8) in the sulcus as the dependent variables. We then tested the direct and indirect effects of age and duration of play. Both age (*β* = 0.367, *p* < 0.001) and duration of play (*β* = 0.424, *p* < 0.001) had a direct effect on tau pathology (Supplementary Table 2). Tau pathology was negatively associated with neuronal density (*β* = −0.335, *p* < 0.001). Overall, this model suggests that the duration of play acts through increased tau pathology to decrease neuronal density within the sulcus (Fig. 5).

## Discussion

Chronic traumatic encephalopathy can be a patchy and focal disease, and its association with gross and microscopic measures of neurodegeneration has not been systematically explored. Here, we show that frontal, hippocampal, hypothalamic, mammillary body, and thalamic atrophy are prominent in high stage CTE. Furthermore, both RHI exposure and increasing CTE stage are associated with neurodegeneration in the dorsolateral frontal cortex, as evidenced by cortical thinning and decreased neuronal density predominantly within the sulcus, as well as synaptic changes in the gyral crest.

The cortical sulcus appears to be uniquely vulnerable to RHI. Computational and physical models that incorporate the gyral architecture of the human brain demonstrate increased strain at the convexities and sulcal depths [27, 30] as well as the formation of cavitation vapor bubbles with the greatest strain at the depths of sulci [37]. The tau pathology in CTE is primarily neuronal and concentrated at the cortical sulcal depths [15]. Furthermore, recent single nuclear RNA sequencing of contact sport athletes with and without CTE shows neuronal loss within the superficial layers at the sulcal depths involving excitatory layer 2/3 CUX2/LAMP5 neurons [16]. Utilizing a larger group of contact/collision sports athletes with and without CTE, we similarly find a sulcal specific pattern of neurodegeneration involving pathways that are both dependent and independent of tau pathology.

Previous MRI studies have assessed different patterns of cortical thinning in AD and frontotemporal dementia (FTD), revealing distinct patterns of regional involvement in AD compared to the frontal and temporal lobes in FTD [29]. Du et al. demonstrated that MRI-based cortical thinning patterns can differentiate between neurodegenerative disease groups [29]. Spotorno et al. investigated cortical thickness in AD and LBD pathology with a direct correlation to postmortem tau burden [66]. Additionally, Mak et al. used PET scans to demonstrate that cortical thinning is associated with tau pathology [43]. Measurement of cortical thickness has proven valuable in detecting cognitive impairment, with evidence that suggests that gray matter loss may underlie cognitive disease [35, 82].

Cortical thinning in youths who had sustained a TBI has also been demonstrated in previous MRI studies [46, 54]. Koerte et al. observed greater cortical thinning in a small group of former professional soccer players compared to non-contact sports athletes, suggesting a potential role of RHI in cortical thinning and cognitive decline [38]. More recently, Albaugh et al. found reduced cortical thickness in the frontal, parietal, and temporal cortices in RHI-exposed ice hockey players, which was associated with post-concussive symptoms [2]. Doughty et al. demonstrated significant cortical thinning, microstructural anomalies, and functional abnormalities in former professional football players that could be distinguished decades after their retirement [28]. Our results support and extend previous MRI findings in patients with CTE [1, 5–7, 74] by demonstrating cortical thinning within the sulcus of the dorsolateral frontal cortex. Future research should focus on distinguishing cortical thickness patterns among neurodegenerative diseases, including CTE, to explore whether in vivo measures can effectively differentiate CTE from other neuropathologies.

Our research builds upon previous studies that have established that AD is characterized by synaptic loss and alterations in synaptic morphology, which may represent compensatory responses to neurodegeneration [23, 24, 42, 45, 61, 64, 65]. Synapse loss, indicating synaptic dysfunction, is considered a reliable marker of cognitive decline in both postmortem and biopsied Alzheimer’s disease brains [23, 64, 65, 72, 79]. Understanding dendritic alterations in neurodegenerative diseases like CTE may provide insights into the underlying mechanisms of cognitive impairment associated with repetitive head injuries. Warling et al. quantified dendrites and dendritic spines of supragranular pyramidal neurons within the frontal and occipital lobes of brain donors with CTE and found increased variability with an overall decrease in dendritic extent in CTE [77]. Furthermore, Braun et al. demonstrated that the mechanical stretching of neurons induces the mislocalization of tau to the dendritic spines, which contributes to synaptic dysfunction [13]. Thus, tau phosphorylation might lead to functional deficits in synaptic function following traumatic brain injuries. Chapman et al. found impaired and reversible synaptic plasticity following high-frequency head impacts in mice [18]. Our previous studies on gene expression networks have shown that genes involved in synaptic pathways are variably altered in Low CTE versus High CTE [39]. This suggests that different processes may be involved depending on the disease progression. In addition, we found sulcal specific network changes in those with a history of RHI and in low stage CTE [19]. Here, we show variation in the levels of α-synuclein and PSD-95 in those with a history of RHI and with CTE, suggesting a continual process of damage and repair that may contribute to disease progression and may have functional consequences. There is likely a dynamic process of synaptic damage and regrowth that underlies resilience and vulnerability to disease and that affects certain regions more than others. Future studies using spatial techniques and super-resolution microscopy should further examine regional vulnerability and the processes that might mediate structural changes in synapses, such as astrocyte and microglia ingestion of synapses, as demonstrated in AD [17, 73].

Finally, cortical thinning has been associated with cognitive decline in Alzheimer’s disease [23–26, 60, 72, 81]. In the context of CTE, cognitive impairments have similarly been observed, with tau pathology in the frontal cortex contributing significantly to cognitive, functional, and neuropsychiatric symptoms [3, 8]. However, tau pathology alone does not fully account for the extent of cognitive decline seen in CTE patients. Our findings suggest that cortical thinning could play an important role in this cognitive decline. In addition, RHI has been associated with a variety of neuropathologies, many of which contribute to functional impairment [63]. Of these, TDP-43 pathology is particularly frequent in CTE [59] and one of the biggest contributors to cognitive impairment [63]. Consistent with this we found in a sensitivity analysis that the presence of TDP-43 inclusions was associated with decreased cortical thickness. While we did not include measures of cognitive decline in our current model due to limitations in statistical power, future research should explore the potential of cortical thinning together with comorbid pathologies as predictive markers for cognitive decline in CTE. Additionally, future studies should include comparisons with other neurodegenerative diseases such as AD and examine markers of cell death and the susceptibility of various cell types, including interneurons. Such studies could provide valuable insights into the multifaceted nature of cognitive impairment following RHI and in CTE and may help identify new diagnostic and therapeutic targets.

### Limitations

There are several limitations to the present study. Brain donors were largely recruited via self-selection or next-of-kin referral, which introduces autopsy-based selection bias that may hinder generalizability. However, inverse probability weighting recently demonstrated that study selection did not significantly affect the relationship between RHI and CTE pathology [40, 55]. The average age of the FHS controls was much older than that of the UNITE brain bank, and PMI was significantly higher in UNITE, given the national catchment area. Although we adjusted for both age and PMI in our analyses, these factors may still contribute to differences. The vast majority of the groups are Caucasian men, further limiting generalizability. Future studies within larger community-based aging cohorts with RHI history, as well as prospective studies, will be necessary to confirm and expand these findings. Finally, synaptic measures were taken from the gyral crest due to the difficulty of sulcal tissue dissections. Future studies, such as super-resolution microscopy, may be necessary to examine alterations in the sulcus [67].

## Conclusions

In those with CTE as the only significant pathology, there is marked cortical atrophy, cortical thinning and decreased neuronal density within the sulcal depth, and altered synaptic protein levels. Contact sports exposure duration was associated with cortical thinning and neuronal loss through tau-dependent and independent mechanisms. These findings may have implications for future MRI and other biomarker studies, where cortical thinning may be useful as a biomarker to detect differential thinning in participants suspected of having CTE. They may also inform assessments of regional-based neurodegeneration following RHI and in CTE.

## Supplementary Information

Below is the link to the electronic supplementary material.

Supplementary file1 (DOCX 79 KB)

## Data Availability

Data used in this study are available from the Boston University Alzheimer’s Disease Center (https://www.bu.edu/alzresearch/information-for-investigators/) and from the authors by request.

## References

[1] Adler CM, DelBello MP, Weber W, Williams M, Duran LRP, Fleck D et al (2018) MRI evidence of neuropathic changes in former college football players. Clin J Sport Med 28:100–105. 10.1097/JSM.0000000000000391 27755011 10.1097/JSM.0000000000000391

[2] Albaugh MD, Orr C, Nickerson JP, Zweber C, Slauterbeck JR, Hipko S et al (2015) postconcussion symptoms are associated with cerebral cortical thickness in healthy collegiate and preparatory school ice hockey players. J Pediatr 166:394-400.e1. 10.1016/j.jpeds.2014.10.016 25454943 10.1016/j.jpeds.2014.10.016

[3] Alosco ML, Barr WB, Banks SJ, Wethe JV, Miller JB, Pulukuri SV et al (2023) Neuropsychological test performance of former American football players. Alzheimers Res Ther 15:1. 10.1186/s13195-022-01147-9 36597138 10.1186/s13195-022-01147-9 PMC9808953

[4] Alosco ML, Cherry JD, Huber BR, Tripodis Y, Baucom Z, Kowall NW et al (2020) Characterizing tau deposition in chronic traumatic encephalopathy (CTE): utility of the McKee CTE staging scheme. Acta Neuropathol 140:495–512. 10.1007/s00401-020-02197-9 32778942 10.1007/s00401-020-02197-9 PMC7914059

[5] Alosco ML, Culhane J, Mez J (2021) Neuroimaging biomarkers of chronic traumatic encephalopathy: targets for the academic memory disorders clinic. Neurotherapeutics 18:772–791. 10.1007/s13311-021-01028-3 33847906 10.1007/s13311-021-01028-3 PMC8423967

[6] Alosco ML, Mian AZ, Buch K, Farris CW, Uretsky M, Tripodis Y et al (2021) Structural MRI profiles and tau correlates of atrophy in autopsy-confirmed CTE. Alzheimers Res Ther 13:193. 10.1186/s13195-021-00928-y 34876229 10.1186/s13195-021-00928-y PMC8653514

[7] Alosco ML, Sugarman MA, Besser LM, Tripodis Y, Martin B, Palmisano JN et al (2018) A clinicopathological investigation of white matter hyperintensities and Alzheimer’s disease neuropathology. J Alzheimers Dis 63:1347–1360. 10.3233/JAD-180017 29843242 10.3233/JAD-180017 PMC6081639

[8] Alosco ML, White M, Bell C, Faheem F, Tripodis Y, Yhang E et al (2024) Cognitive, functional, and neuropsychiatric correlates of regional tau pathology in autopsy-confirmed chronic traumatic encephalopathy. Mol Neurodegener 19:10. 10.1186/s13024-023-00697-2 38317248 10.1186/s13024-023-00697-2 PMC10845638

[9] Apostolova LG, Steiner CA, Akopyan GG, Dutton RA, Hayashi KM, Toga AW et al (2007) Three-dimensional gray matter atrophy mapping in mild cognitive impairment and mild Alzheimer disease. Arch Neurol 64:1489–1495. 10.1001/archneur.64.10.1489 17923632 10.1001/archneur.64.10.1489 PMC3197839

[10] Bieniek KF, Cairns NJ, Crary JF, Dickson DW, Folkerth RD, Keene CD et al (2021) The second NINDS/NIBIB consensus meeting to define neuropathological criteria for the diagnosis of chronic traumatic encephalopathy. J Neuropathol Exp Neurol 80:210–219. 10.1093/jnen/nlab001 33611507 10.1093/jnen/nlab001 PMC7899277

[11] Bieniek KF, Ross OA, Cormier KA, Walton RL, Soto-Ortolaza A, Johnston AE et al (2015) Chronic traumatic encephalopathy pathology in a neurodegenerative disorders brain bank. Acta Neuropathol 130:877–889. 10.1007/s00401-015-1502-4 26518018 10.1007/s00401-015-1502-4 PMC4655127

[12] Bigio EH, Vono MB, Satumtira S, Adamson J, Sontag E, Hynan LS et al (2001) Cortical synapse loss in progressive supranuclear palsy. J Neuropathol Exp Neurol 60:403–410. 10.1093/jnen/60.5.403 11379815 10.1093/jnen/60.5.403

[13] Braun NJ, Yao KR, Alford PW, Liao D (2020) Mechanical injuries of neurons induce tau mislocalization to dendritic spines and tau-dependent synaptic dysfunction. Proc Natl Acad Sci USA 117:29069. 10.1073/pnas.2008306117 33139536 10.1073/pnas.2008306117 PMC7682580

[14] Bruce HJ, Tripodis Y, McClean M, Korell M, Tanner CM, Contreras B et al (2023) American football play and parkinson disease among men. JAMA Netw Open 6:e2328644. 10.1001/jamanetworkopen.2023.28644 37566412 10.1001/jamanetworkopen.2023.28644 PMC10422187

[15] Butler MLMD, Dixon E, Stein TD, Alvarez VE, Huber B, Buckland ME et al (2022) Tau pathology in chronic traumatic encephalopathy is primarily neuronal. J Neuropathol Exp Neurol 81:773. 10.1093/jnen/nlac065 35903039 10.1093/jnen/nlac065 PMC9487650

[16] Butler MLMD, Pervaiz N, Ypsilantis P, Wang Y, Cammasola Breda J, Mazzilli S et al (2024) Repetitive head impacts induce neuronal loss and neuroinflammation in young athletes. bioRxiv. 10.1101/2024.03.26.586815 39605380 10.1101/2024.11.08.622719 PMC11601221

[17] Cardozo PL, de Lima IBQ, Maciel EMA, Silva NC, Dobransky T, Ribeiro FM (2019) Synaptic elimination in neurological disorders. CN 17:1071–1095. 10.2174/1570159X17666190603170511 10.2174/1570159X17666190603170511 PMC7052824 31161981

[18] Chapman DP, Power SD, Vicini S, Ryan TJ, Burns MP (2024) Amnesia after repeated head impact is caused by impaired synaptic plasticity in the memory engram. J Neurosci 44:e1560232024. 10.1523/JNEUROSCI.1560-23.2024 38228367 10.1523/JNEUROSCI.1560-23.2024 PMC10883615

[19] Cherry JD, Agus F, Dixon E, Huber B, Alvarez VE, Mez J et al (2021) Differential gene expression in the cortical sulcus compared to the gyral crest within the early stages of chronic traumatic encephalopathy. Free Neuropathol. 10.17879/FREENEUROPATHOLOGY-2021-3453 34485990 10.17879/freeneuropathology-2021-3453 PMC8415801

[20] Clare R, King VG, Wirenfeldt M, Vinters HV (2010) Synapse loss in dementias. J Neurosci Res 88:2083–2090. 10.1002/jnr.22392 20533377 10.1002/jnr.22392 PMC3068914

[21] Coleman PD, Flood DG (1987) Neuron numbers and dendritic extent in normal aging and Alzheimer’s disease. Neurobiol Aging 8:521–545. 10.1016/0197-4580(87)90127-8 3323927 10.1016/0197-4580(87)90127-8

[22] Davies CA, Mann DMA, Sumpter PQ, Yates PO (1987) A quantitative morphometric analysis of the neuronal and synaptic content of the frontal and temporal cortex in patients with Alzheimer’s disease. J Neurol Sci 78:151–164. 10.1016/0022-510X(87)90057-8 3572454 10.1016/0022-510x(87)90057-8

[23] DeKosky ST, Scheff SW (1990) Synapse loss in frontal cortex biopsies in Alzheimer’s disease: correlation with cognitive severity. Ann Neurol 27:457–464. 10.1002/ana.410270502 2360787 10.1002/ana.410270502

[24] DeKosky ST, Scheff SW, Styren SD (1996) Structural correlates of cognition in dementia: quantification and assessment of synapse change. Neurodegeneration 5:417–421. 10.1006/neur.1996.0056 9117556 10.1006/neur.1996.0056

[25] Dickerson BC, Bakkour A, Salat DH, Feczko E, Pacheco J, Greve DN et al (2009) The cortical signature of alzheimer’s disease: regionally specific cortical thinning relates to symptom severity in very mild to Mild AD dementia and is detectable in asymptomatic amyloid-positive individuals. Cereb Cortex 19:497–510. 10.1093/cercor/bhn113 18632739 10.1093/cercor/bhn113 PMC2638813

[26] Dickerson BC, Wolk DA (2012) MRI cortical thickness biomarker predicts AD-like CSF and cognitive decline in normal adults. Neurology 78:84–90. 10.1212/WNL.0b013e31823efc6c 22189451 10.1212/WNL.0b013e31823efc6c PMC3466670

[27] Donat CK, Yanez Lopez M, Sastre M, Baxan N, Goldfinger M, Seeamber R et al (2021) From biomechanics to pathology: predicting axonal injury from patterns of strain after traumatic brain injury. Brain 144:70–91. 10.1093/brain/awaa336 33454735 10.1093/brain/awaa336 PMC7990483

[28] Doughty M, Danielli E, Boshra R, Ruiter KI, Minuzzi L, Connolly JF et al (2023) Years of play alter MRI measures of brain health in former Canadian Football League athletes: a pilot study. J Concussion 7:20597002231200372. 10.1177/20597002231200372

[29] Du A-T, Schuff N, Kramer JH, Rosen HJ, Gorno-Tempini ML, Rankin K et al (2006) Different regional patterns of cortical thinning in Alzheimer’s disease and frontotemporal dementia. Brain 130:1159–1166. 10.1093/brain/awm016 10.1093/brain/awm016 PMC1853284 17353226

[30] Ghajari M, Hellyer PJ, Sharp DJ (2017) Computational modelling of traumatic brain injury predicts the location of chronic traumatic encephalopathy pathology. Brain 140:333–343. 10.1093/brain/aww317 28043957 10.1093/brain/aww317 PMC5278309

[31] Gittins R, Harrison PJ (2004) Neuronal density, size and shape in the human anterior cingulate cortex: a comparison of Nissl and NeuN staining. Brain Res Bull 63:155–160. 10.1016/j.brainresbull.2004.02.005 15130705 10.1016/j.brainresbull.2004.02.005

[32] Gómez-Isla T, Price JL, McKeel DW Jr, Morris JC, Growdon JH, Hyman BT (1996) Profound loss of layer ii entorhinal cortex neurons occurs in very mild Alzheimer’s disease. J Neurosci 16:4491–4500. 10.1523/JNEUROSCI.16-14-04491.1996 8699259 10.1523/JNEUROSCI.16-14-04491.1996 PMC6578866

[33] Gottschall PE, Ajmo JM, Eakin AK, Howell MD, Mehta H, Bailey LA (2010) Panel of synaptic protein ELISAs for evaluating neurological phenotype. Exp Brain Res 201:885–893. 10.1007/s00221-010-2182-x 20169337 10.1007/s00221-010-2182-x PMC2864526

[34] Gunzler D, Chen T, Wu P, Zhang H (2013) Introduction to mediation analysis with structural equation modeling. Shanghai Arch Psychiatry 25:390–394. 10.3969/j.issn.1002-0829.2013.06.009 24991183 10.3969/j.issn.1002-0829.2013.06.009 PMC4054581

[35] Hwang KS, Beyer MK, Green AE, Chung C, Thompson PM, Janvin C et al (2013) Mapping cortical atrophy in parkinson’s disease patients with dementia. J Parkinsons Dis 3:69–76. 10.3233/JPD-120151 23938313 10.3233/JPD-120151 PMC4018208

[36] Jamjoom AAB, Rhodes J, Andrews PJD, Grant SGN (2021) The synapse in traumatic brain injury. Brain 144:18–31. 10.1093/brain/awaa321 33186462 10.1093/brain/awaa321 PMC7880663

[37] Kerwin J, Yücesoy A, Vidhate S, Dávila-Montero BM, Van Orman JL, Pence TJ et al (2022) Sulcal cavitation in linear head acceleration: possible correlation with chronic traumatic encephalopathy. Front Neurol 13:832370. 10.3389/fneur.2022.832370 35295830 10.3389/fneur.2022.832370 PMC8918564

[38] Koerte IK, Mayinger M, Muehlmann M, Kaufmann D, Lin AP, Steffinger D et al (2016) Cortical thinning in former professional soccer players. Brain Imaging Behav 10:792–798. 10.1007/s11682-015-9442-0 26286826 10.1007/s11682-015-9442-0

[39] Labadorf A, Agus F, Aytan N, Cherry J, Mez J, McKee A et al (2023) Inflammation and neuronal gene expression changes differ in early versus late chronic traumatic encephalopathy brain. BMC Med Genomics 16:49. 10.1186/s12920-023-01471-5 36895005 10.1186/s12920-023-01471-5 PMC9996917

[40] LeClair J, Weuve J, Fox MP, Mez J, Alosco ML, Nowinski C et al (2022) Selection bias analysis supports dose-response relationship between level of american football play and chronic traumatic encephalopathy diagnosis. Am J Epidemiol. 10.1093/aje/kwac075 35434739 10.1093/aje/kwac075 PMC9989358

[41] Lepeta K, Lourenco MV, Schweitzer BC, Martino Adami PV, Banerjee P, Catuara-Solarz S et al (2016) Synaptopathies: synaptic dysfunction in neurological disorders—a review from students to students. J Neurochem 138:785–805. 10.1111/jnc.13713 27333343 10.1111/jnc.13713 PMC5095804

[42] Li K, Wei Q, Liu F-F, Hu F, Xie A, Zhu L-Q et al (2018) Synaptic dysfunction in Alzheimer’s disease: Aβ, tau, and epigenetic alterations. Mol Neurobiol. 10.1007/s12035-017-0533-3 28456942 10.1007/s12035-017-0533-3

[43] Mak E, Bethlehem RAI, Romero-Garcia R, Cervenka S, Rittman T, Gabel S et al (2018) In vivo coupling of tau pathology and cortical thinning in Alzheimer’s disease. Alzheimers Dement 10:678–687. 10.1016/j.dadm.2018.08.005 10.1016/j.dadm.2018.08.005 PMC6222030 30426064

[44] Marksteiner J, Kaufmann WA, Gurka P, Humpel C (2002) Synaptic proteins in Alzheimer’s disease. J Mol Neurosci 18:53–63. 10.1385/JMN:18:1-2:53 11931350 10.1385/JMN:18:1-2:53

[45] Masliah E, Mallory M, Alford M, DeTeresa R, Hansen LA, McKeel DW et al (2001) Altered expression of synaptic proteins occurs early during progression of Alzheimer’s disease. Neurology 56:127–129. 10.1212/wnl.56.1.127 11148253 10.1212/wnl.56.1.127

[46] McCauley SR, Wilde EA, Merkley TL, Schnelle KP, Bigler ED, Hunter JV et al (2010) Patterns of cortical thinning in relation to event-based prospective memory performance three months after moderate to severe traumatic brain injury in children. Dev Neuropsychol 35:318–332. 10.1080/87565641003696866 20446135 10.1080/87565641003696866 PMC3061405

[47] McKee AC, Alosco ML, Huber BR (2016) Repetitive head impacts and chronic traumatic encephalopathy. Neurosurg Clin N Am 27:529–535. 10.1016/j.nec.2016.05.009 27637402 10.1016/j.nec.2016.05.009 PMC5028120

[48] McKee AC, Cairns NJ, Dickson DW, Folkerth RD, Keene CD, Litvan I et al (2016) The first NINDS/NIBIB consensus meeting to define neuropathological criteria for the diagnosis of chronic traumatic encephalopathy. Acta Neuropathol 131:75–86. 10.1007/s00401-015-1515-z 26667418 10.1007/s00401-015-1515-z PMC4698281

[49] McKee AC, Cantu RC, Nowinski CJ, Hedley-Whyte ET, Gavett BE, Budson AE et al (2009) Chronic traumatic encephalopathy in athletes: progressive tauopathy after repetitive head injury. J Neuropathol Exp Neurol 68:709–735. 10.1097/NEN.0b013e3181a9d503 19535999 10.1097/NEN.0b013e3181a9d503 PMC2945234

[50] McKee AC, Daneshvar DH, Alvarez VE, Stein TD (2014) The neuropathology of sport. Acta Neuropathol 127:29–51. 10.1007/s00401-013-1230-6 24366527 10.1007/s00401-013-1230-6 PMC4255282

[51] McKee AC, Mez J, Abdolmohammadi B, Butler M, Huber BR, Uretsky M et al (2023) Neuropathologic and clinical findings in young contact sport athletes exposed to repetitive head impacts. JAMA Neurol 80:1037–1050. 10.1001/jamaneurol.2023.2907 37639244 10.1001/jamaneurol.2023.2907 PMC10463175

[52] McKee AC, Stein TD, Kiernan PT, Alvarez VE (2015) The neuropathology of chronic traumatic encephalopathy: CTE neuropathology. Brain Pathol 25:350–364. 10.1111/bpa.12248 25904048 10.1111/bpa.12248 PMC4526170

[53] McKee AC, Stein TD, Nowinski CJ, Stern RA, Daneshvar DH, Alvarez VE et al (2013) The spectrum of disease in chronic traumatic encephalopathy. Brain 136:43–64. 10.1093/brain/aws307 23208308 10.1093/brain/aws307 PMC3624697

[54] Merkley TL, Bigler ED, Wilde EA, McCauley SR, Hunter JV, Levin HS (2008) Short communication: diffuse changes in cortical thickness in pediatric moderate-to-severe traumatic brain injury. J Neurotrauma 25:1343–1345. 10.1089/neu.2008.0615 19061377 10.1089/neu.2008.0615 PMC2747789

[55] Mez J, Daneshvar DH, Abdolmohammadi B, Chua AS, Alosco ML, Kiernan PT et al (2020) Duration of American football play and chronic traumatic encephalopathy. Ann Neurol 87:116–131. 10.1002/ana.25611 31589352 10.1002/ana.25611 PMC6973077

[56] Mez J, Daneshvar DH, Kiernan PT, Abdolmohammadi B, Alvarez VE, Huber BR et al (2017) Clinicopathological evaluation of chronic traumatic encephalopathy in players of American football. JAMA 318:360. 10.1001/jama.2017.8334 28742910 10.1001/jama.2017.8334 PMC5807097

[57] Mez J, Solomon TM, Daneshvar DH, Murphy L, Kiernan PT, Montenigro PH et al (2015) Assessing clinicopathological correlation in chronic traumatic encephalopathy: rationale and methods for the UNITE study. Alz Res Therapy 7:62. 10.1186/s13195-015-0148-8 10.1186/s13195-015-0148-8 PMC4601147 26455775

[58] Montenigro PH, Baugh CM, Daneshvar DH, Mez J, Budson AE, Au R et al (2014) Clinical subtypes of chronic traumatic encephalopathy: literature review and proposed research diagnostic criteria for traumatic encephalopathy syndrome. Alz Res Therapy 6:68. 10.1186/s13195-014-0068-z 10.1186/s13195-014-0068-z PMC4288217 25580160

[59] Nicks R, Clement NF, Alvarez VE, Tripodis Y, Baucom ZH, Huber BR et al (2023) Repetitive head impacts and chronic traumatic encephalopathy are associated with TDP-43 inclusions and hippocampal sclerosis. Acta Neuropathol 145:395–408. 10.1007/s00401-023-02539-3 36681782 10.1007/s00401-023-02539-3 PMC11360224

[60] Ossenkoppele R, Smith R, Ohlsson T, Strandberg O, Mattsson N, Insel PS et al (2019) Associations between tau, Aβ, and cortical thickness with cognition in Alzheimer disease. Neurology 92:e601–e612. 10.1212/WNL.0000000000006875 30626656 10.1212/WNL.0000000000006875 PMC6382060

[61] Reddy PH, Mani G, Park BS, Jacques J, Murdoch G, Whetsell W et al (2005) Differential loss of synaptic proteins in Alzheimer’s disease: implications for synaptic dysfunction. J Alzheimers Dis 7:103–117. 10.3233/jad-2005-7203 15851848 10.3233/jad-2005-7203

[62] Regeur L (2000) Increasing loss of brain tissue with increasing dementia: a stereological study of post-mortem brains from elderly females. Eur J Neurol 7:47–54. 10.1046/j.1468-1331.2000.00017.x 10809914 10.1046/j.1468-1331.2000.00017.x

[63] Saltiel N, Tripodis Y, Menzin T, Olaniyan A, Baucom Z, Yhang E et al (2024) Relative contributions of mixed pathologies to cognitive and functional symptoms in brain donors exposed to repetitive head impacts. Ann Neurol 95:314–324. 10.1002/ana.26823 37921042 10.1002/ana.26823 PMC10842014

[64] Scheff SW, DeKosky ST, Price DA (1990) Quantitative assessment of cortical synaptic density in Alzheimer’s disease. Neurobiol Aging 11:29–37. 10.1016/0197-4580(90)90059-9 2325814 10.1016/0197-4580(90)90059-9

[65] Scheff SW, Price DA (1993) Synapse loss in the temporal lobe in Alzheimer’s disease. Ann Neurol 33:190–199. 10.1002/ana.410330209 8434881 10.1002/ana.410330209

[66] Spotorno N, Coughlin DG, Olm CA, Wolk D, Vaishnavi SN, Shaw LM et al (2020) Tau pathology associates with in vivo cortical thinning in Lewy body disorders. Ann Clin Transl Neurol 7:2342–2355. 10.1002/acn3.51183 33108692 10.1002/acn3.51183 PMC7732256

[67] Standring OJ, Friedberg J, Tripodis Y, Chua AS, Cherry JD, Alvarez VE et al (2019) Contact sport participation and chronic traumatic encephalopathy are associated with altered severity and distribution of cerebral amyloid angiopathy. Acta Neuropathol 138:401–413. 10.1007/s00401-019-02031-x 31183671 10.1007/s00401-019-02031-x PMC6689453

[68] Stathas S, Alvarez VE, Xia W, Nicks R, Meng G, Daley S et al (2021) Tau phosphorylation sites serine202 and serine396 are differently altered in chronic traumatic encephalopathy and Alzheimer’s disease. Alzheimer’s Dement. 10.1002/alz.12502 10.1002/alz.12502 PMC9160206 34854540

[69] Stein TD, Montenigro PH, Alvarez VE, Xia W, Crary JF, Tripodis Y et al (2015) Beta-amyloid deposition in chronic traumatic encephalopathy. Acta Neuropathol 130:21–34. 10.1007/s00401-015-1435-y 25943889 10.1007/s00401-015-1435-y PMC4529056

[70] Stern RA, Daneshvar DH, Baugh CM, Seichepine DR, Montenigro PH, Riley DO et al (2013) Clinical presentation of chronic traumatic encephalopathy. Neurology 81:1122–1129. 10.1212/WNL.0b013e3182a55f7f 23966253 10.1212/WNL.0b013e3182a55f7f PMC3795597

[71] Templeton GF (2011) A two-step approach for transforming continuous variables to normal: implications and recommendations for IS research. CAIS. 10.1705/1CAIS.02804

[72] Terry RD, Masliah E, Salmon DP, Butters N, DeTeresa R, Hill R et al (1991) Physical basis of cognitive alterations in Alzheimer’s disease: synapse loss is the major correlate of cognitive impairment. Ann Neurol 30:572–580. 10.1002/ana.410300410 1789684 10.1002/ana.410300410

[73] Tzioras M, Daniels MJD, Davies C, Baxter P, King D, McKay S et al (2023) Human astrocytes and microglia show augmented ingestion of synapses in Alzheimer’s disease via MFG-E8. Cell Rep Med 4:101175. 10.1016/j.xcrm.2023.101175 37652017 10.1016/j.xcrm.2023.101175 PMC10518633

[74] Uretsky M, Bouix S, Killiany RJ, Tripodis Y, Martin B, Palmisano J et al (2022) Association between antemortem FLAIR white matter hyperintensities and neuropathology in brain donors exposed to repetitive head impacts. Neurology 98:e27–e39. 10.1212/WNL.0000000000013012 34819338 10.1212/WNL.0000000000013012 PMC8726571

[75] Vallortigara J, Whitfield D, Quelch W, Alghamdi A, Howlett D, Hortobágyi T et al (2016) Decreased levels of VAMP2 and monomeric alpha-synuclein correlate with duration of dementia. J Alzheimers Dis 50:101–110. 10.3233/JAD-150707 26639969 10.3233/JAD-150707

[76] Vonsattel JPG, Del Amaya MP, Keller CE (2008) Twenty-first century brain banking. Processing brains for research: the Columbia University methods. Acta Neuropathol 115:509–532. 10.1007/s00401-007-0311-9 17985145 10.1007/s00401-007-0311-9 PMC2292479

[77] Warling A, Uchida R, Shin H, Dodelson C, Garcia ME, Shea-Shumsky NB et al (2021) Putative dendritic correlates of chronic traumatic encephalopathy: a preliminary quantitative Golgi exploration. J Comp Neurol 529:1308–1326. 10.1002/cne.25022 32869318 10.1002/cne.25022 PMC8554981

[78] Whitwell JL, Shiung MM, Przybelski S, Weigand SD, Knopman DS, Boeve BF et al (2008) MRI patterns of atrophy associated with progression to AD in amnestic Mild cognitive impairment. Neurology 70:512–520. 10.1212/01.wnl.0000280575.77437.a2 17898323 10.1212/01.wnl.0000280575.77437.a2 PMC2734138

[79] de Wilde MC, Overk CR, Sijben JW, Masliah E (2016) Meta-analysis of synaptic pathology in Alzheimer’s disease reveals selective molecular vesicular machinery vulnerability. Alzheimers Dement 12:633–644. 10.1016/j.jalz.2015.12.005 26776762 10.1016/j.jalz.2015.12.005 PMC5058345

[80] Woodworth DC, Nguyen KM, Kawas CH, Corrada MMM, Sajjadi SA (2023) Gross measures of neurodegeneration from autopsy explain as much variance in dementia severity as measures from histology. Alzheimer’s Dement 19:e079477. 10.1002/alz.079477

[81] Yoon EJ, Lee J-Y, Kwak S, Kim YK (2023) Mild behavioral impairment linked to progression to Alzheimer’s disease and cortical thinning in amnestic mild cognitive impairment. Front Aging Neurosci 14:1051621. 10.3389/fnagi.2022.1051621 36688162 10.3389/fnagi.2022.1051621 PMC9846631

[82] Zarei M, Ibarretxe-Bilbao N, Compta Y, Hough M, Junque C, Bargallo N et al (2013) Cortical thinning is associated with disease stages and dementia in Parkinson’s disease. J Neurol Neurosurg Psychiatry 84:875–882. 10.1136/jnnp-2012-304126 23463873 10.1136/jnnp-2012-304126 PMC3717586

[83] Zimmerman KA, Kim J, Karton C, Lochhead L, Sharp DJ, Hoshizaki T et al (2021) Player position in American football influences the magnitude of mechanical strains produced in the location of chronic traumatic encephalopathy pathology: a computational modelling study. J Biomech 118:110256. 10.1016/j.jbiomech.2021.110256 33545573 10.1016/j.jbiomech.2021.110256 PMC7612336

