# Supplementary material for: Neurodegeneration in the cortical sulcus is a feature of chronic traumatic encephalopathy and associated with repetitive head impacts
Source: Acta Neuropathol. 2024 Dec 6;148(1):79. doi: 10.1007/s00401-024-02833-8 (PMC11624223; doi:10.1007/s00401-024-02833-8)
Supplement: Supplementary file 1 — Supplementary file1 (DOCX 79 KB) [file 401_2024_2833_MOESM1_ESM.docx]

| **Supplementary Table 1:**  Mean tau pathology scores in cortical brain regions across groups | | | | | | |
| --- | --- | --- | --- | --- | --- | --- |
| **Characteristic** | | Control  (n=52) | RHI  (n = 48) | Low CTE  (n = 49) | High CTE  (n = 88) | P |
| **Tau pathology burden, semiquantitative 0-3**  **Mean (SEM)** | |  |  |  |  |  |
| Middle frontal | | 0.08 (0.11) | 0.43 (0.11) | 1.16 (0.11) | 2.35 (0.09) | <0.001*^bcdef^ |
| Inferior orbitofrontal | | 0.06 (0.24) | 0.22 (0.13) | 0.66 (0.13) | 1.78 (0.10) | <0.001*^cef^ |
| Superior temporal | | 0.34 (0.13) | 0.31 (0.13) | 0.86 (0.13) | 2.28 (0.10) | <0.001*^cef^ |
| Inferior parietal | | 0.03 (0.13) | 0.14 (0.13) | 0.73 (0.13) | 1.81 (0.10) | <0.001*^bcdef^ |
|  | *****Analysis of Variance, with post-hoc least significant difference statistical testing p<0.05 as follows:  b: Low CTE is significantly different than Control, with p < 0.05  c: High CTE is significantly different than Control, with p < 0.05  d: Low CTE is significantly different than RHI, with p < 0.05  e: High CTE is significantly different than RHI, with p < 0.05  f: High CTE is significantly different than Low CTE, with p < 0.05  CTE: Chronic Traumatic Encephalopathy; FHS: Framingham Heart Study; LOC: Loss of Consciousness; PMI: Post-Mortem Interval; RHI: Repetitive Head Impacts; SEM: Standard Error of Mean; | | | | | |

**Supplementary Table 2**: Mediation analysis, with neuronal density in the sulcus (NeuN), and Tau pathology (AT8 Sulcus) as the independent variables. Neuronal density as measured by NeuN, with repetitive head impacts, Tau pathology (AT8 sulcus), and age as predictors.

|  |  | |  |  | |
| --- | --- | --- | --- | --- | --- |
|  | Effect | |  |  |  |
|  | NeuN | Tau pathology (AT8 sulcus) |  |  |  |
| RHI exposure (yrs) |  |  |  |  |  |
| Est. Std | -0.080 | 0.424 |  |  |  |
| Std error | 0.166 | 0.062 |  |  |  |
| z-value | -0.482 | 6.709 |  |  |  |
| p(>\|z\|) | 0.630 | **<0.001** |  |  |  |
| Age |  |  |  |  |  |
| Est. Std | 0.135 | 0.367 |  |  |  |
| Std error | 0.162 | 0.065 |  |  |  |
| z-value | 0.837 | 5.666 |  |  |  |
| p(>\|z\|) | 0.403 | **<0.001** |  |  |  |
| Tau pathology (AT8 sulcus) |  |  |  |  |  |
| Est. Std | -0.335 | X |  |  |  |
| Std error | 0.134 |  |  |  |  |
| z-value | -2.492 |  |  |  |  |
| p(>\|z\|) | **0.013** |  |  |  |  |

p<0.05 in bold
Est. Std, estimated standard effect; CTE, chronic traumatic encephalopathy; RHI, repetitive head impacts

**Supplementary Figure:**

**Supplementary Figure. Differences in cortical thickness and sulcus/crest ratio between groups without age adjustment.** a) Cortical thickness within the sulcus was significantly different between groups (p=0.017, analysis of variance). Post-hoc pairwise comparisons showed that cortical thickness was reduced in High CTE compared to the control (p=0.022) and RHI (p=0.004) groups. b) Ratio of cortical thickness sulcus/crest was significantly different between groups (p<0.001, analysis of variance). Post-hoc comparisons showed that RHI (p=0.004), Low CTE (p=0.001), and High CTE (p<0.001) groups all had lower sulcus/crest ratios than the control group. *p<0.05; **p<0.01
